# Risk Factors and Awareness of Bone Fragility in Inflammatory Bowel Disease in Taiwan: A Cross-Sectional Study

**DOI:** 10.3390/biomedicines13030638

**Published:** 2025-03-05

**Authors:** Yao-Wei Kuo, Chia-Jung Kuo, Puo-Hsien Le, Ming-Ling Chang, Cheng-Yu Lin, Chen-Ming Hsu, Wei-Pin Lin, Chun-Wei Chen, Wey-Ran Lin, Yu-Pin Ho, Ming-Yao Su, Cheng-Tang Chiu

**Affiliations:** 1 Department of Gastroenterology and Hepatology, Chang Gung Memorial Hospital, Linkou 333423, Taiwan; a87098@cgmh.org.tw (Y.-W.K.); b9005031@cgmh.org.tw (P.-H.L.); minglingc@cgmh.org.tw (M.-L.C.); 8805035@cgmh.org.tw (C.-Y.L.); hsu3060e@cgmh.org.tw (C.-M.H.); weipin@cgmh.org.tw (W.-P.L.); 8902088@cgmh.org.tw (C.-W.C.); t12360@cgmh.org.tw (W.-R.L.); hoyupin@cgmh.org.tw (Y.-P.H.); ctchiu0508@cgmh.org.tw (C.-T.C.); 2 College of Medicine, Chang Gung University, Taoyuan 333001, Taiwan; doctorsu@cgmh.org.tw; 3 Chang Gung Microbiota Therapy Center, Taoyuan 333423, Taiwan; 4 Chang Gung Inflammatory Bowel Diseases Center, Linkou Chang Gung Memorial Hospital, Taoyuan 333423, Taiwan; 5 Department of Gastroenterology and Hepatology, New Taipei Municipal Tu Cheng Hospital (Built and Operated by Change Gung Medical Foundation), New Taipei City 236017, Taiwan; 6 Taiwan Association for the Study of Intestinal Disease, Taoyuan 333423, Taiwan

**Keywords:** Crohn’s disease, ulcerative colitis, inflammatory bowel disease, osteoporosis, awareness, knowledge, physical activity

## Abstract

**Background/Objectives:** Patients with inflammatory bowel disease (IBD) are at a higher risk of developing bone disorders. Awareness and understanding of the disease are crucial for prevention and early diagnosis. Currently, there is no research on the risk factors and knowledge of bone fragility in the population with IBD in Taiwan. This study aimed to evaluate the risk factors and self-assessed knowledge levels of bone health among patients with IBD in Taiwan. **Methods:** This single-center cross-sectional study included 59 adult patients. Clinical data, blood tests, bone mineral density (BMD), T-score, Z-score, and questionnaires covering self-assessed knowledge, fracture risks, and physical activity were assessed. The patients were divided into normal and low BMD groups. **Results:** Of all participants, eighteen (30.5%) had low BMD: six (10.2%) had BMD below the expected range, ten (16.9%) had osteopenia, and two (3.4%) had osteoporosis. Vitamin D insufficiency and deficiency were observed in 26.3% and 66.6% of the patients, respectively. According to multivariate analysis, age and sex hormone deficiency are strongly associated with low BMD. Educational interventions significantly improved the patients’ self-assessed knowledge levels. **Conclusions:** Age and sex hormone deficiency are significant factors contributing to low BMD in IBD patients. Not only women but also men with IBD who had symptoms of hypogonadism are at high risk for low BMD. Educational interventions improve self-assessment knowledge regarding the relationship between IBD and bone health.

## 1. Introduction

Inflammatory bowel diseases (IBD), including Crohn’s disease (CD) and ulcerative colitis (UC), are chronic inflammatory conditions. Both UC and CD can cause extra-intestinal symptoms, affecting the eyes, skin, liver, bones, and joints in 25–40% of patients [1]. Musculoskeletal disorders, including osteoporosis, are among the most common extraintestinal complications in IBD patients [2].

Osteoporosis is a bone disorder caused by an imbalance between bone resorption and formation, which leads to decreased bone strength and a higher risk of fractures. Bone disorders can also affect both morbidity and mortality [3]. Patients with IBD are at a higher risk for osteoporosis due to progressive symptoms such as systemic inflammation, nutrient deficiencies (e.g., vitamin D), hypogonadism, and treatments such as corticosteroid therapy or surgical resection [4]. A previous study in Taiwan estimated that the incidence of osteoporosis was 40% higher in the IBD cohort than in the non-IBD cohort (7.16 vs. 5.13 per 1000 person-years) [5]. In addition, a recent systematic review analyzing data from 3661 IBD patients and 12,789 healthy individuals reported osteoporosis prevalence rates of 2–16% in the IBD group (7–15% for Crohn’s disease and 2–9% for ulcerative colitis) and 3–10% in the healthy group [6].

Although various treatment options for osteoporosis are available and widely accepted, prevention remains the most effective way to avoid complications [7]. To prevent and manage metabolic bone diseases in IBD patients at an early stage, it is crucial to establish clear diagnostic and treatment protocols, reducing the risk of fractures. Additionally, strengthening interdisciplinary cooperation is essential to ensure that patients receive timely and appropriate treatment.

Previous studies have shown that young female students, adults, and older women often lack sufficient knowledge of the disease [8,9]. This lack of awareness of key risk factors and preventive measures can hinder the adoption of a healthier lifestyle and the effective use of preventive services. Although IBD patients are at an increased risk of bone fragility, no studies have specifically evaluated their awareness of the disease or the effects of educational interventions. This study aims to explore the risk factors of low bone mineral density and assess bone health awareness in IBD patients in Taiwan.

## 2. Materials and Methods

This single-center cross-sectional study was conducted at Chang Gung Memorial Hospital, Linkou Branch, from October 2023 to June 2024. The study was approved by the Ethics Review Board of Chang Gung Memorial Hospital (IRB No. 202202123B0). The inclusion criteria were as follows: (1) adult patients aged ≥ 18 years and (2) confirmed diagnosis of IBD, including UC or CD. IBD was diagnosed based on established clinical, endoscopic, and histological criteria in accordance with the European Crohn’s and Colitis Organization (ECCO) guidelines. The exclusion criteria were as follows: (1) age < 18 years; (2) pregnancy; (3) the presence of other conditions which may affecting bone mineral density (BMD), including diabetes, liver diseases, chronic kidney diseases, chronic obstructive pulmonary disease, thyroid diseases, rheumatoid arthritis, active malignancy, other serious ongoing disease; and (4) lack of written informed consent to participate in the study. All patients in the study received treatment based on the consensus guidelines of the Taiwan Society of IBD and the European Crohn’s and Colitis Organization tailored to their clinical condition.

### 2.1. Data Extraction

Clinical data were collected through patient interviews and a review of medical records. Information gathered included age, sex, smoking status, body mass index (BMI), physical activity, IBD-related details (disease subtype, duration, prior surgeries, and prescribed medications), and additional nutritional supplements (vitamin D and calcium). After the initial interview, venous blood samples were collected for laboratory analysis, including complete blood count, C-reactive protein, albumin, ferritin, liver and kidney function tests, calcium, phosphate, intact parathyroid hormone, vitamin B12, folate, and vitamin D levels.

### 2.2. Bone Mineral Density (BMD) Assessment

According to the International Society of Clinical Densitometry (ISCD) guidelines [10], a BMD below the expected range for age is defined as a Z-score of <−2 standard deviations (SD) at the hip or spine for premenopausal women and men aged < 50 years. For other patients, a T-score is used, with a T-score <−2.5 SD indicating osteoporosis and a T-score between −2.5 and −1 SD indicating osteopenia. Patients were classified into two groups based on the following criteria: those with normal BMD and those with low BMD (Z-score <−2 SD for premenopausal women and men under 50 years, or T-score <−1 SD for other patients).

### 2.3. Structured Questionnaire

This study included two questionnaires: the Chinese version of the One-Minute Osteoporosis Risk Check Questionnaire and the self-assessed knowledge questionnaire [11]. Completing all the questionnaires took approximately 20 min. The risk of osteoporosis was assessed using the Chinese version of the One-Minute Osteoporosis Risk Test, which was translated and adapted by the Taiwanese Osteoporosis Association based on the original version developed by the International Osteoporosis Foundation (IOF). The test evaluated 19 key risk factors with a total score of 19. Patients who answered “YES” to any sex hormone-related questions from Questions 16 to 19 were classified as having sex hormone deficiency. We also included questions about physical activity. The questionnaire asks participants if they engage in regular exercise. If they answer yes, their physical activity level is assessed and categorized as high, moderate, or low. A high level is defined as performing vigorous-intensity activities (e.g., running, fast swimming, aerobic exercises) at least three days per week. A moderate level is defined as engaging in moderate-intensity activities (e.g., hiking, swimming at a regular pace, cycling) on at least five days per week. A low level refers to physical activity that does not meet the criteria for moderate or high levels.

A self-designed questionnaire with six questions was used to assess participants’ knowledge about osteoporosis and IBD. Participants were asked to rate their knowledge about various aspects of osteoporosis, such as its connection to IBD, risk factors (e.g., steroid use and malabsorption), and their anxiety regarding osteoporosis. The answer options ranged from “totally unaware” to “totally understand” on a 10-point scale. After completing the questionnaire, participants were provided with educational materials about the association between IBD and osteoporosis, including the risks, potential adverse events, and importance of prevention. Following the educational session, a post-test was conducted using the same questionnaire to assess any changes in knowledge.

### 2.4. Statistical Analysis

Descriptive statistics for continuous variables are presented as mean ± SD. For skewed distributions, categorical variables were expressed as proportions, and binary variables as percentages. *p*-values were determined using a two-sample *t*-test or Mann–Whitney U test for continuous variables and a chi-square or Fisher’s exact test for categorical variables. One-way analysis of variance (ANOVA) was used to assess statistical significance between the means of three or more independent groups. Binary logistic regression was used to identify independent predictors of low BMD. Statistical significance was set at *p* < 0.05. Subjects with missing data are excluded. Statistical analyses were conducted using SPSS for Windows (version 20.0; IBM Corp, Armonk, NY, USA), and graphs were generated using GraphPad Prism version 10.0.0 Windows; GraphPad Software, Boston, MA, USA, www.graphpad.com, accessed on 6 November 2024.).

### 2.5. Use of OpenAI ChatGPT-4o

ChatGPT-4o (GPT-4o OpenAI’s large-scale language-generation model) was used to check the language and grammar in the article. The authors reviewed, edited, and revised the text generated by ChatGPT to match their preferences and take full responsibility for the final content of the publication.

## 3. Results

**Figure 1:**
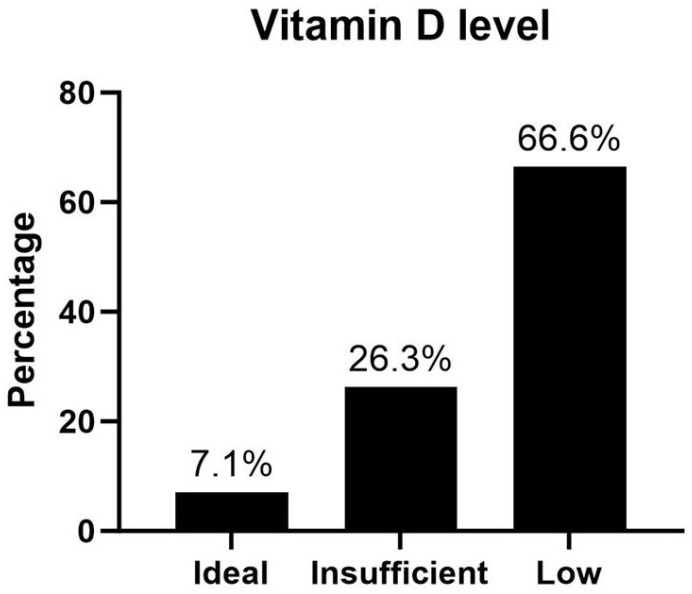
Vitamin D levels in patients with inflammatory bowel disease.

Initially, 60 patients were enrolled in the study. However, one patient was diagnosed with a different type of colitis, and two declined BMD assessment. Finally, 59 patients were included, with 18 (30.5%) diagnosed with UC and 41 (69.5%) with CD. The demographic and clinical characteristics of patients are shown in Table 1. A total of 43 patients (72.9%) were male, while 16 patients (27.1%) were female. The median BMI was 23.5 kg/m^2^ (IQR 21–26.9), and the median age was 43 years (IQR 34–55). The mean disease duration was 96.16 ± 56.56 months. A total of eight (13.6%) patients had a history of small bowel resection, and twenty-three (39%) received steroid treatment at study inclusion. Additionally, 23 (39%) patients were on immunosuppressants, and 30 (50.8%) were on biologic therapy. More than half of the patients (33 patients, 55.9%) engaged in high physical activity. In total, eighteen (30.5%) and six (10.2%) patients were under low and moderate physical activity. The median vitamin D level was 15.30 ng/mL (IQR 9.75–23.05). Only 7.1% of patients had an ideal vitamin D level (>30 ng/mL), 26.3% had insufficient levels (20–30 ng/mL), and 66.6% (<20 ng/mL) had low levels (Figure 1). Among all participants, eighteen patients (30.5%) were found to have low BMD, with six patients (10.2%) classified as having BMD below expected levels, ten patients (16.9%) diagnosed with osteopenia, and two patients (3.4%) identified as having osteoporosis. Additionally, we used the One-Minute Osteoporosis Risk Questionnaire from the International Osteoporosis Foundation (IOF) in our study. The overall results are presented in Supplementary Table S1.

### 3.1. Characteristics of Low BMD and Its Risk Factors

As displayed in Table 2, patients with low BMD were older than those with normal BMD (mean age: 41.58 ± 12.61 vs. 52.61 ± 13.95 years, *p* = 0.004). No statistical differences were found between the two groups in terms of sex, BMI, IBD subtype, disease duration, history of small bowel resection, or steroid use. Over half of the patients in both groups engaged in high physical activity (60.5% vs. 55.6%, *p* = 0.915). There were no differences in nutritional supplement use, including calcium (38.5% vs. 33.3%, *p* = 0.775) and vitamin D (38.5% vs. 22.2%, *p* = 0.365) between the two groups. In venous blood profiles, only the WBC count showed statistical significance, although the median WBC levels were within the normal range in both groups (median: 7.4 1000/uL [IQR 6.3–8.3] vs. 5.8 1000/uL [IQR 5.3–7.25], *p* = 0.003).

**Figure 2:**
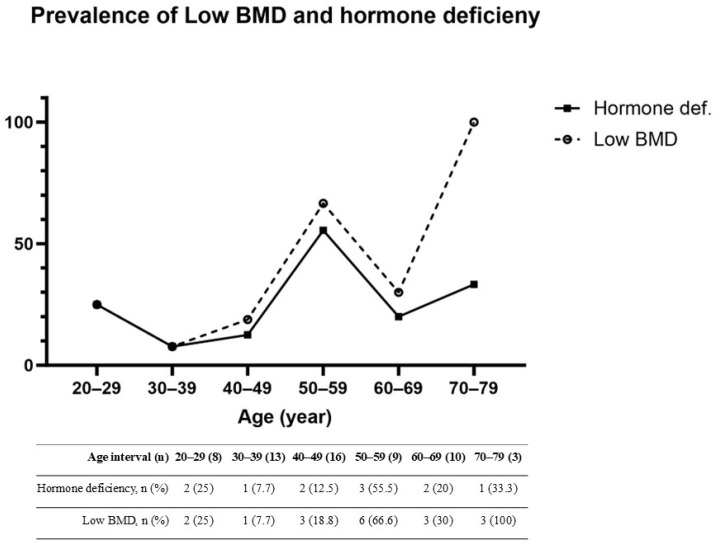
The relationship between the prevalence of sex hormone deficiency and low BMD across different age intervals. BMD, bone mineral density.

In terms of risk factors from the One-Minute Osteoporosis Risk Check Questionnaire, the low BMD group had a significantly higher proportion of sex hormone deficiency (12.8% vs. 44.4%, *p* = 0.015) (Table 3). Sex hormone deficiency strongly correlated with the prevalence of low BMD (Figure 2). We conducted two-step logistic regression analysis. In the univariate model, sex hormone deficiency (OR 5.440; 95% CI 1.45–2.38; *p* = 0.012) and age (OR 1.063; 95% CI 1.016–1.112; *p* = 0.008) were associated with a higher risk of low BMD. In the multivariate model, sex hormone deficiency (OR 4.56; 95% CI 1.12–18.55; *p* = 0.034) and age (OR 1.058; 95% CI 1.009–1.108; *p* = 0.019) continued to show a significant influence. All the data are presented in Table 4.

### 3.2. Self-Assessed Knowledge About Osteoporosis and IBD

The self-assessment of knowledge questionnaire was designed to evaluate self-reported knowledge about osteoporosis and its association with IBD. Table 5 presents the questions and data collected before and after the educational intervention. In the pretest, questions about “the need for bone mineral density testing with long-term steroid use” (score: 2.36 ± 2.86) and “the impact of steroids on bone mineral density” (score: 2.37 ± 2.79) received the lowest scores. On the other hand, question about “the link between steroid use and osteoporosis” achieved the highest score (score: 4.06 ± 3.69). After the educational intervention, most questions showed a significant improvement in knowledge scores, from questions 1 to 5. However, there was no significant improvement in scores related to anxiety about the risk of osteoporosis after education intervention (score: 4.01 ± 3.04 vs. 4.96 ± 3.28; *p* = 0.07).

## 4. Discussion

In this cross-sectional study of IBD patients, we found that age and sex hormone deficiency were associated with low BMD. Age has always been a non-modified risk factor for poor bone health [12]. Age has also been identified as an important risk factor for the IBD population. In a recent systematic review and meta-analysis, increased age was associated with decreased BMD [6]. Although age and low BMD are significantly correlated, the strength of this association varies across age groups. In our study, low BMD gradually increased with age. There was a steep increase in the prevalence of low BMD after age 50 years, with a sharp rise in low BMD prevalence after this age. This finding is consistent with previous research, where age showed only a minor effect on bone health in younger adults aged 20–50 [13].

Hypogonadism is a well-known factor affecting bone health. It is the primary physiological change in postmenopausal women with low BMD and the most common cause of osteoporosis in men [14,15,16]. Sex hormone deficiency significantly impacts bone health by disrupting the balance between bone resorption and formation, leading to bone loss and increased fracture risk. Estrogen plays a critical role in maintaining bone density in both sexes, primarily by inhibiting osteoclast activity and promoting osteoblast survival [17]. Its deficiency, as seen in postmenopausal women or men with hypogonadism, results in increased bone remodeling, where bone resorption outpaces bone formation [18]. This imbalance leads to microarchitectural deterioration and decreased bone strength. In men, testosterone also contributes to bone health through direct androgen receptor activation and its conversion to estrogen via aromatase [19]. Testosterone deficiency, as seen in hypogonadism or during androgen deprivation therapy, impairs both bone formation and resorption. Additionally, sex hormone deficiency influences the secretion of cytokines such as RANKL and OPG, which regulate osteoclastogenesis and affect the pathway critical for osteoblast differentiation [20]. These mechanisms collectively result in reduced bone mass and heightened fracture susceptibility. In females, hypogonadism can be identified by symptoms such as amenorrhea, menopause, or medical history, such as ovarian surgery [21]. In males, the gold standard for diagnosing hypogonadism is to identify clinical androgen deficiency and confirm it using biochemical testing [22,23]. Although our study focused on symptoms such as impotence and low libido, previous research shows that sexual complaints are often linked to low testosterone levels [23,24]. Hypogonadism is not uncommon in IBD patients. It can result from inflammation or cytokines that affect the reproductive system. Other contributing factors include impaired ovarian and testicular function, undernutrition, and the impact of chronic steroid use on gonadotropin secretion [25]. In IBD patients, studies have reported a higher prevalence of testosterone deficiency in men than in controls [8,15,26,27]. Additionally, IBD in women is associated with an earlier onset of menopause [28]. In our study, the prevalence of low BMD and sex hormone deficiency were highly aligned with each other. Research on the link between hypogonadism and osteoporosis in IBD patients remains scarce [29]. Current guidelines suggest BMD screening for IBD patients with conventional osteoporosis risk factors as for the general population [7]. However, the role of hypogonadism in IBD may be more significant and may warrant special attention. Notably, previous studies have identified women as a high-risk group for low BMD. In our study, however, men constituted the majority of patients with low BMD. Male patients with IBD exhibiting symptoms of sex hormone deficiency may represent a high-risk group for low BMD and should be evaluated accordingly.

Compared with UC patients, CD patients are generally considered to have a higher risk of osteoporosis; however, this trend was not observed in our study. A 2020 systematic review of the prevalence of osteoporosis and low BMD in population-based studies identified CD as one of the major risk factors for low BMD and osteoporosis [6]. Similarly, a population-based study in Taiwan reported a higher risk of osteoporosis in patients with CD but not in patients with UC [5]. The increased risk of osteoporosis in CD patients has been attributed to factors such as small bowel disease or resection, smoking, and higher corticosteroid use [6]. In our study, there were no significant differences in smoking or steroid use between the CD and UC groups. All eight patients with prior small bowel resection were diagnosed with CD. Two of these patients were found to have low BMD—one with BMD below expected levels and one with osteopenia. In the subgroup analysis of patients with UC and CD, there were no significant differences in clinical characteristics between the two groups. However, despite borderline significance in the subgroup analysis, UC patients tended to be older than CD patients, which may explain the similar prevalence of low BMD in both groups (median: 54-year-old [IQR 36.0–65.2] vs. 42-year-old [IQR 32.5–52.0], *p* = 0.074) (Supplementary Table S2).

Physical activity showed no obvious impact on BMD in the present study, possibly because of the high prevalence of low vitamin D levels. It has been well established that physical activity increases BMD and prevents osteoporosis in both the general population and IBD patients [30,31,32,33,34]. Although exercise is recommended for patients with IBD, their physical activity was generally significantly lower in previous studies [12,35,36,37,38]. Previous studies that used the International Physical Activity Questionnaire (IPAQ) to evaluate physical activity in patients with IBD reported that the proportion engaging in high-level physical activity ranged from 0% to 27.2% [36,38,39]. In our study, 40.7% of patients engaged in high-level physical activity. A recent population-based study in an Asian country suggested that the protective effect of physical activity becomes less apparent under conditions of low vitamin D levels [40]. Despite varying cutoff points for defining low vitamin D levels, previous meta-analyses estimated a prevalence of 38.1–57.7% in CD and 31.6% in UC [14]. In our study, only 7.1% of the patients had ideal vitamin D levels, which may mask the beneficial impact of physical activity on bone health. A recent study on vitamin D levels in the IBD population in central Taiwan reported the prevalence of vitamin D insufficiency and deficiency to be 41.5% and 42.5%, respectively [41]. Our study showed a relatively higher prevalence, which may be linked to the location of our research center in northern Taiwan. Previous studies have identified sunlight exposure as a key factor for vitamin D deficiency. The subtropical climate of northern Taiwan may contribute to higher rates of vitamin D deficiency [42,43].

Educational intervention improves self-assessed knowledge of osteoporosis in patients with IBD. Adequate awareness and knowledge of osteoporosis are crucial for fostering self-efficacy, encouraging lifestyle changes, and enabling early diagnosis. Beyond general knowledge, “self-assessment knowledge” also plays a positive role in patients’ self-management [44]. Currently, there is a lack of literature on bone health and awareness in patients with IBD. In our study, the educational intervention improved self-assessment knowledge; however, it had no impact on anxiety in patients with IBD. Further research is needed to evaluate whether an improvement in self-assessment knowledge through an educational intervention can influence patient behavior and promote long-term benefits, such as improved BMD and reduced fracture risk.

Our study had several limitations. First, the limited number of patients in the outcome measures may compromise the statistical power and generalizability of the findings. Second, it was challenging to accurately assess the intake of substances such as calcium and vitamins and the cumulative dose of steroids prior to study enrollment. Third, the evaluation of sex hormone deficiency relied on patient history and sex-related symptoms, without measuring serum estrogen or testosterone levels. Fourth, our study used a non-standardized self-assessment to evaluate knowledge about the association between IBD and osteoporosis. Using a standardized knowledge scale may better reflect the actual situation. Nonetheless, our study is the first to address critical risk factors for low BMD in patients with IBD in Taiwan and to examine the potential impact of hypogonadism and osteoporosis in this population. We also highlighted the potential benefits of educational interventions in improving bone health among patients with IBD. Further research is necessary to determine whether such interventions can effectively modify patient behavior, enhance bone health, and reduce fracture rates. More studies are needed to better understand the specific roles of hypogonadism and osteoporosis in patients with IBD.

## 5. Conclusions

In conclusion, age and sex hormone deficiency are significant factors contributing to low BMD in IBD patients. Not only women but also men with IBD who show symptoms of sex hormone deficiency are at high risk for low BMD. Educational interventions improve self-assessment knowledge regarding the relationship between IBD and bone health.

## Figures and Tables

**Table 1:** Overview of demographic and clinical characteristics in IBD patients. SD, standard deviation; IQR, interquartile range; CD, Crohn’s disease; BMI, body mass index; 5-ASA, 5-aminosalicylic acid; TNF, tumor necrosis factor; IL, interleukin; CRP, C-reactive protein; AST, aspartate transaminase; ALT, alanine transaminase; Ca, calcium; BMD, bone mineral density.

	Overall (n = 59)
Men, n (%)	43 (72.9)
Age (year), median (IQR)	43 (34–55)
BMI (kg/m^2^), median (IQR)	23.5 (21–26.9)
Smoking, n (%)	6 (10.2)
Disease type-CD, n (%)	41 (69.5)
Disease duration (months), mean (SD)	96.16 (56.56)
Previous small bowel resection, n (%)	8 (13.6)
5-ASA use, n (%)	48 (81.4)
Corticosteroid use, n (%)	23 (39)
Immunosuppressant use, n (%)	23 (39)
Biologic use, n (%)	30 (50.8)
Nil, n (%)	29 (49.2)
Anti-TNF, n (%)	15 (25.4)
Anti-IL12/23, n (%)	10 (16.9)
Anti-integrin, n (%)	5 (8.5)
Physical activity	
Low, n (%)	18 (30.5)
Moderate, n (%)	15 (25.4)
High, n (%)	24 (40.7)
Nutritional supplement	
Ca, n (%)	22 (37.3)
Vitamin D, n (%)	19 (32.2)
WBC (1000/uL), mean (SD)	7.12 (1.89)
Hemoglobin (g/dl), median (IQR)	13.6 (11.6–14.3)
CRP (mg/L), median (IQR)	2.16 (0.63–4.60)
Albumin (g/dL), median (IQR)	4.11 (3.81–4.47)
Ferritin (ng/mL), median (IQR)	68.3 (30.2–164.5)
Creatinine (mg/dL), median (IQR)	0.79 (0.68–0.96)
AST (U/L), median (IQR)	17.0 (12.0–25.0)
ALT (U/L), median (IQR)	18.0 (18.0–26.0)
Ca (mg/dl), median (IQR)	9.0 (8.70–9.27)
Inorganic *p* (mg/dL), mean (SD)	3.40 (0.54)
Intact-PTH (pg/mL), median (IQR)	35.8 (27.65–52.80)
Vitamin B12 (pg/mL), median (IQR)	382.5 (236.7–521.7)
Folic acid (ng/mL), median (IQR)	9.00 (6.00–18.20)
Vitamin D (ng/mL), median (IQR)	15.30 (9.75–23.05)
BMD	
Hip (g/cm^2^), mean (SD)	0.70 (0.52)
Lumber (g/cm^2^), mean (SD)	1.09 (1.24)
T score	
Hip, mean (SD)	−1.00 (1.03)
Lumber, mean (SD)	−1.02 (1.27)
Z score	
Hip, mean (SD)	−0.73 (0.96)
Lumber, mean (SD)	−1.02 (1.27)
Low BMD, n (%)	18 (30.5)
BMD below expected, n (%)	6 (10.2)
Osteopenia, n (%)	10 (16.9)
Osteoporosis, n (%)	2 (3.4)

**Table 2:** Demographic, clinical, laboratory, and parameters in IBD patients, according to the presence or absence of low BMD. SD, standard deviation; IQR, interquartile range; CD, Crohn’s disease; BMI, body mass index; 5-ASA, 5-aminosalicylic acid; TNF, tumor necrosis factor; IL, interleukin; CRP, C-reactive protein; AST, aspartate transaminase; ALT, alanine transaminase; Ca, calcium; BMD, bone mineral density. * *p* < 0.05; ** *p* <0.01.

	Normal BMD (n = 39)	Low BMD (n = 18)	*p* Value
Men, n (%)	27 (69.2)	15 (83.3)	0.342
Age (year), mean (SD)	41.58 (12.61)	52.61 (13.95)	0.004 **
BMI (kg/m^2^), mean (SD)	24.28 (4.35)	24.06 (4.03)	0.857
Smoking, n (%)	5 (12.8)	0 (0)	0.168
Disease type-CD, n (%)	30 (76.9)	10 (55.6)	0.126
Disease duration (months), median (IQR)	91.97 (51.33–128.27)	91.7 (46.59–121.46)	0.925
Previous small bowel resection, n (%)	6 (15.4)	2 (11.1)	1
5-ASA use, n (%)	31 (79.5)	16 (88.9)	0.323
Current steroid use, n (%)	16 (41.0)	6 (33.3)	0.771
Immunosuppressant use, n (%)	14 (35)	9 (52.9)	0.388
Biologic use, n (%)	21 (53.8)	8 (44.4)	0.576
Physical activity			0.936
Low, n (%)	12 (31.6)	6 (33.3)	
Moderate, n (%)	9 (23.7)	5 (27.8)	
High, n (%)	17 (44.7)	7 (38.9)	
Nutritional supplement			
Ca, n (%)	15 (38.5)	6 (33.3)	0.775
Vit D, n (%)	15 (38.5)	4 (22.2)	0.365
WBC (1000/uL), median (IQR)	7.4 (6.3–8.3)	5.8 (5.3–7.25)	0.03 *
Hemoglobin (g/dl), median (IQR)	13.3 (10.9–14.2)	13.85 (12.4–14.3)	0.304
CRP (mg/L), median (IQR)	2.36 (1.00–3.74)	1.18 (0.33–7.47)	0.456
Albumin (g/dL), mean (SD)	4.05 (0.56)	4.14 (0.41)	0.612
Ferritin (ng/mL), median (IQR)	48 (13.9–159)	127.5 (78–244.25)	0.078
Creatinine (mg/dL), median (IQR)	0.78 (0.68–0.90)	0.89 (0.73–1.03)	0.131
AST (U/L), median (IQR)	16 (12.0–23.0)	19 (13.2–31.0)	0.405
ALT (U/L), median (IQR)	19 (12.0–27.0)	15 (11.0–28.5)	0.808
Ca (mg/dl), median (IQR)	9 (8.75–9.35)	8.75 (8.65–9.05)	0.096
Inorganic *p* (mg/dL), median (IQR)	3.40 (3.15–3.70)	3.20 (2.77–3.92)	0.614
Intact-PTH (pg/mL), median (IQR)	36.9 (28.70–52.55)	35.4 (24.97–56.87)	0.877
Vitamin B12 (pg/mL), mean (SD)	379 (204.8)	482 (321.0)	0.183
Folic acid (ng/mL), mean (SD)	10.75 (9.26)	16.21 (9.99)	0.081
Vitamin D (ng/mL), mean (SD)	16.91 (11.14)	17.11 (7.05)	0.946

**Table 3:** Result of One-Minute Osteoporosis Risk Check Questionnaire according to presence of low BMD; BMD, bone mineral density. ^1^ Sex hormone deficiency defined as patients who answered “YES” to any sex hormone-related questions from Questions 16 to 19 were classified as having sex hormone deficiency. * *p* < 0.05.

			Normal BMD (n = 40)	Low BMD (n = 17)	*p* Value
Q1:	Parents diagnosed with osteoporosis? n (%)		8 (20.5)	3 (16.7)	1.000
Q2:	Parents have a stooped back (dowager’s hump)? n (%)		4 (10.3)	2 (11.1)	1.000
Q3:	Are you 40 years old or older? n (%)		21 (53.8)	14 (77.8)	0.142
Q4:	Have you broken a bone from a minor fall as an adult? n (%)		3 (7.7)	1 (5.6)	1.000
Q5:	Do you fall often (more than once last year) or fear falling due to frailty? n (%)		5 (12.8)	0 (0)	0.168
Q6:	Have you lost more than 3 cm (over 1 inch) in height since age 40? n (%)		3 (7.7)	2 (11.1)	0.646
Q7:	Is your body mass index (BMI) below 19 kg/m^2^? n (%)		7 (21.2)	5 (31.3)	0.492
Q8:	Have you taken corticosteroid tablets for over 3 months in a row? n (%)		25 (61.4)	12 (66.7)	1.000
Q9:	Have you been diagnosed with rheumatoid arthritis? n (%)		0 (0)	2 (11.1)	0.096
Q10:	Have you ever had hyperthyroidism or hyperparathyroidism? n (%)		0 (0)	0 (0)	.
Q11:	Do you drink alcohol regularly beyond safe limits (over two units a day)? n (%)		1 (2.6)	0 (0)	1.000
Q12:	Do you currently smoke, or have you ever smoked? n (%)		6 (15.4)	1 (5.6)	0.413
Q13:	Do you perform less than 30 min of physical activity daily (housework, gardening, walking, running etc.)? n (%)		7 (17.9)	7 (38.9)	0.107
Q14:	Do you avoid milk or dairy and not take calcium supplements? n (%)		22 (57.9)	14 (77.8)	0.233
Q15:	Do you spend less than 10 min outdoors daily without vitamin D supplements? n (%)		16 (41)	11 (64.7)	0.147
Q16–Q19: Sex hormone deficiency ^1^, n (%)			5 (12.8)	8 (44.4)	0.015 *
	Q16:	Did you reach menopause before age 45? n/n (%)	0/11 (0)	0/3 (0)	
	Q17:	Have your periods ever stopped for 12 months or more (not due to pregnancy, menopause, or hysterectomy)? n/n (%)	1/11 (9)	2/3 (0)	
	Q18:	Were your ovaries removed before age 50 without hormone replacement therapy? n/n (%)	0/11 (0)	0/3 (0)	
	Q19:	Have you ever experienced impotence, low libido, or other low testosterone symptoms? n/n (%)	4/28 (14.3)	6/15 (40)	

**Table 4:** Relationship between sex hormone deficiency, age, and low BMD in univariate and multivariate logistic regression; CI, confidence interval.

Univariate Logistic Regression.			Multivariate Logistic Regression	
	**Odd Ratio (95% CI)**	*p* Value	Odd Ratio (95% CI)	*p* Value
Sex hormone deficiency	5.440 (1.45–2.38)	0.012	4.56 (1.12–18.55)	0.034
Age	1.063 (1.016–1.112)	0.008	1.058 (1.009–1.108)	0.019

**Table 5:** Difference in self-assessed knowledge scores of osteoporosis and IBD before and after the educational intervention.; IBD, inflammatory bowel disease.

		Pretest (Mean ± SD)	Post-Test (Mean ± SD)	*p* Value
Q1:	How well do you understand the link between IBD and osteoporosis?	3.25 ± 3.31	6.89 ± 3.18	<0.001
Q2:	How well do you understand the link between steroid use and osteoporosis?	4.06 ± 3.69	6.65 ± 3.36	<0.001
Q3:	How well do you understand the need for bone mineral density testing in long-term steroid users?	2.36 ± 2.86	6.87 ± 3.11	<0.001
Q4:	How well do you understand the impact of steroids on bone quality and density?	2.37 ± 2.79	6.34 ± 3.42	<0.001
Q5:	How well do you understand how malabsorption in IBD can affect bone health?	3.81 ± 3.40	6.89 ± 3.18	<0.001
Q6:	How anxious do you feel about the overall risk of osteoporosis in IBD?	4.01 ± 3.04	4.96 ± 3.28	0.07

## Data Availability

The data supporting the findings of this study are not publicly available due to ethical considerations but can be obtained from the corresponding author upon reasonable request.

## Supplementary Materials

The following supporting information can be downloaded at https://www.mdpi.com/article/10.3390/biomedicines13030638/s1, Table S1: Overall result of One-minute osteoporosis risk questionnaire in IBD patients.; Table S2: Subgroup analysis of demographic, clinical, laboratory, and BMD parameters in IBD patients.

## Abbreviations

The following abbreviations are used in this manuscript:

5-ASA	5-aminosalicylic acid
AST	Aspartate transaminase
ALT	Alanine transaminase
BMD	Bone mineral density
BMI	Body mass index
CD	Crohn’s disease
CI	Confidence interval
CRP	C-reactive protein
ECCO	European Crohn’s and Colitis Organization
IBD	Inflammatory bowel disease
ISCD	International Society of Clinical Densitometry
IOF	International Osteoporosis Foundation
IPAQ	International Physical Activity Questionnaire
IL	Interleukin
SD	Standard deviations
TNF	Tumor necrosis factor
UC	Ulcerative colitis
