# Supplementary material for: Risk Factors and Awareness of Bone Fragility in Inflammatory Bowel Disease in Taiwan: A Cross-Sectional Study
Source: Biomedicines. 2025 Mar 5;13(3):638. doi: 10.3390/biomedicines13030638 (PMC11940530; doi:10.3390/biomedicines13030638)
Supplement: Supplementary file 1 [file biomedicines-13-00638-s001.zip › Supplementary_Table_S1.pdf]

Table S1. Overall result of One-minute osteoporosis risk questionnaire in IBD patients.

		Overall (n=58)
Q1:	Parents diagnosed with osteoporosis? n (%)	11 (18.6)
Q2:	Parents have a stooped back (dowager's hump)? n (%)	6 (10.2)
Q3:	Are you 40 years old or older? n (%)	35 (59.3)
Q4:	Have you broken a bone from a minor fall as an adult? n (%)	5 (8.5)
Q5:	Do you fall often (more than once last year) or fear falling due to frailty? n (%)	6 (10.2)
Q6:	Have you lost more than 3 cm (over 1 inch) in height since age 40? n (%)	5 (8.5)
Q7:	Is your Body Mass Index (BMI) below 19 kg/m^2? n (%)	12 (20.3)
Q8:	Have you taken corticosteroid tablets for over 3 months in a row? n (%)	38 (64.4)
Q9:	Have you been diagnosed with rheumatoid arthritis? n (%)	2 (3.4)
Q10:	Have you ever had hyperthyroidism or hyperparathyroidism? n (%)	1 (1.7)
Q11:	Do you drink alcohol regularly beyond safe limits (over two units a day)? n (%) n (%)	1 (1.7)
Q12:	Do you currently smoke, or have you ever smoked? n (%)	7 (11.9)
Q13:	Do you do less than 30 minutes of physical activity daily (housework, gardening, walking, running etc.)? n (%)	14 (23.7)
Q14:	Do you avoid milk or dairy and not take calcium supplements? n (%)	36 (61)
Q15:	Do you spend less than 10 minutes outdoors daily without vitamin D supplements? n (%)	28 (47.5)
**For women**		
Q16:	Did you reach menopause before age 45? n (%)	0 (0)
Q17:	Have your periods ever stopped for 12 months or more (not due to pregnancy, menopause, or hysterectomy)? n (%)	3 (5.1)
Q18:	Were your ovaries removed before age 50 without Hormone Replacement Therapy? n (%)	0 (0)
**For men**		
Q19:	Have you ever experienced impotence, low libido, or other low testosterone symptoms? n (%)	10 (16.9)
