# Supplementary material for: Risk Factors and Awareness of Bone Fragility in Inflammatory Bowel Disease in Taiwan: A Cross-Sectional Study
Source: Biomedicines. 2025 Mar 5;13(3):638. doi: 10.3390/biomedicines13030638 (PMC11940530; doi:10.3390/biomedicines13030638)
Supplement: Supplementary file 1 [file biomedicines-13-00638-s001.zip › Supplementary_Table_S2.pdf]

Table S2. Subgroup analysis of demographic, clinical, laboratory, and BMD parameters in IBD patients.

	**UC (n = 18)**	**CD (n = 41)**	**P value**
Men, n (%)	13 (72.2)	30 (73.2)	1.000
Age (year), median (IQR)	54 (36.0-65.2)	42 (32.5-52.0)	0.074
BMI (kg/m2), median (IQR)			0.398
Smoking, n (%)	0 (0)	6 (14.6)	0.164
Disease duration (months), median (IQR)			0.517
Previous small bowel resection, n (%)	0 (0)	8 (19.5)	0.092
5-ASA use, n (%)	14 (77.8)	31 (75.6)	1.000
Current steroid use, n (%)	5 (27.8)	18 (43.9)	0.385
Immunosuppressant use, n (%)	4 (22.2)	19 (46.3)	0.093
Biologic use, n (%)	6 (33.3)	24 (58.5)	0.095
Physical activity			0.877
Low, n (%)	6 (33.3)	12 (30.8)	
Moderate, n (%)	4 (22.2)	11 (28.2)	
High, n (%)	8 (44.4)	16 (41.0)	
Nutritional supplement			
Ca, n (%)	9 (50.0)	13 (32.5)	0.249
Vit D, n (%)	5 (27.8)	14 (35.0)	0.764
Low BMD, n (%)	8 (47.1)	10 (25.0)	0.126
